# Effectiveness and Safety of Botulinum Toxin Type A in Treatment of Restless Legs Syndrome: A Systematic Review and Meta-Analysis

**DOI:** 10.3390/healthcare9111538

**Published:** 2021-11-11

**Authors:** Yu-Chi Su, Yao-Hong Guo, Chung-Lun Liao, Yu-Ching Lin

**Affiliations:** 1 National Cheng Kung University Hospital, College of Medicine, National Cheng Kung University, Tainan 70428, Taiwan; zac850429@gmail.com; 2 Department of Physical Medicine and Rehabilitation, National Cheng Kung University Hospital, College of Medicine, National Cheng Kung University, Tainan 70428, Taiwan; patchguo@gmail.com; 3 School of Medicine, College of Medicine, National Cheng Kung University, Tainan 70428, Taiwan; i54046314@gs.ncku.edu.tw; 4 Department of Physical Medicine and Rehabilitation, College of Medicine, National Cheng Kung University, Tainan 70428, Taiwan

**Keywords:** botulinum toxin, restless legs syndrome, systematic review, meta-analysis

## Abstract

Our study aimed to investigate the effectiveness and safety of botulinum toxin type A in patients with restless legs syndrome. We searched electronic databases, including PubMed, Cochrane Library, and Web of Science, up to 12 June 2021, for published articles. We enrolled randomized controlled clinical trials and non-randomized controlled studies involving patients with restless legs syndrome who were treated with botulinum toxin. Quality assessment was performed using the Cochrane risk of bias tool and Joanna Briggs Institute Critical Appraisal Checklist for Quasi-Experimental Studies. As for the results, we included four articles comprising 62 participants, two studies were randomized controlled trials. Improvement in International Restless Legs Syndrome Study Group (IRLSSG) rating scale was observed in three studies. Adverse events were temporary and self-limited. Meta-analyses were performed, including the two randomized controlled trials with 27 participants. Compared with placebo, botulinum toxin injection significantly reduced scores of IRLSSG rating scale (SMD, −0.819, 95% confidence interval [CI], −1.377 to −0.262). A total of 11.8% (95% CI, 0.7–72.4%) of patients reported at least one adverse event. In conclusion, botulinum toxin injection may relieve restless legs syndrome related symptoms. However, decisive conclusions cannot be drawn because of the small number of patients included in our meta-analysis. Large-scale, randomized controlled trials are warranted to discover the optimal dose, safety, and long-term effect of intervention with botulinum toxin type A for patients with restless legs syndrome.

## 1. Introduction

Restless legs syndrome (RLS) is a sleep-related movement disorder characterized by an unpleasant urge to move the lower limbs. The prevalence of RLS varies by region, ethnicity, sex, and age, ranging from 5–15% [1]. Its pathophysiology remains unclear. Criteria for the diagnosis of RLS include the International Restless Legs Syndrome Study Group (IRLSSG) and International Classification of Sleep Disorders, Third Edition (ICSD-3) [2,3]. The ICSD-3 criteria require distress and associated sleep disturbance, which is different from the IRLSSG consensus [2]. As for the measurement of disease severity for RLS, the IRLSSG rating scale (IRLS) was proposed. It assesses a range of RLS related symptoms and their impact on patients’ mood and daily life, and it has been proved reliable, valid, and responsive in clinical trials [4].

Pharmacologic therapies for RLS include dopamine agonists, alpha-2-delta calcium channel ligands, and iron supplements [5]. Several non-pharmacological interventions have been proposed to treat RLS, such as repetitive transcranial magnetic stimulation, exercise, compression devices, counter-strain manipulation, infrared therapy, and acupuncture; however, they have relatively low quality of evidence [6]. Despite the treatments mentioned above, about 45% of patients remain unchanged or are worse [7,8]. Therefore, finding alternative therapies that complement the conventional treatments would be beneficial.

In patients with RLS, hyperalgesia in the leg was revealed in previous studies [9,10,11]. Besides, 21.4% to 61% of RLS patients have described their symptoms as painful [12]. Additionally, botulinum neurotoxins (BoNT) may relieve hyperalgesia and pain probably through blocking the release of pain mediators in the peripheral terminals, dorsal root ganglia and spinal cord neurons [13,14,15,16]. Hence, we may expect improvements in patients with RLS after BoNT treatment. However, controversies exist in the effect of BoNT in RLS [17,18,19,20,21,22,23], and no articles have evaluated this topic systemically.

The objective of this article is to investigate the effectiveness of BoNT in RLS by conducting a systematic review and meta-analysis of published articles in PubMed, Web of Science and Cochrane Library between inception and 12 June 2021. Our hypothesis was that in patients with RLS, treatment with BoNT would reduce the IRLS score. We also aimed to delineate the possible factors affecting the treatment effect of BoNT, including dosage, injection site, and commercial forms of BoNT. We included randomized controlled trials and non-randomized controlled studies.

## 2. Materials and Methods

We conducted this systematic review according to the Preferred Reporting Items for Systematic Review and Meta-Analysis (PRISMA) guidelines [24]. We registered the review with the International Prospective Register of Systematic Reviews (PROSPERO), ID: CRD42021278137.

### 2.1. Eligibility Criteria

We selected randomized controlled clinical trials and non-randomized controlled studies involving patients with RLS treated with botulinum toxin type A. Inclusion criteria were studies with patients with RLS treated with botulinum toxin type A, and the follow-up time should be at least 4 weeks after injection. Studies of any duration of intervention were included. Studies with control groups were required to be treated with placebo to be eligible for analysis. Exclusion criteria were articles published in language other than English, case reports and conference proceedings due to high possibility of publication bias.

### 2.2. Search Strategy

We searched PubMed, Cochrane Central Register of Controlled Trials, and Web of Science for papers with language restricted to English. We retrieved the literature using the medical subject heading terms: “restless legs syndrome” AND “botulinum toxin”. The search time was from inception to the present time, and the final search was on 12 June 2021 (see File S1. for full search strategy).

### 2.3. Study Selection and Data Extraction

Three authors (YCS, YHG, and CLL) removed duplicate articles, reviewed the titles and abstracts of pertinent studies independently. If consensus could not be made following discussion, they sought the opinion of the senior author (YCL). We made use of a data sheet to collect data from the recruited articles, which included first author, year of publication, characteristics of participants, commercial brands of botulinum toxin, dosage of botulinum toxin, dilution method, total sessions of injection, site of injection, methods of guidance, comparative regimen, and clinical outcomes. The authors were contacted as necessary to resolve any uncertainties.

### 2.4. Quality Assessment

First, we graded the level of evidence of the included studies according to the Oxford Centre for Evidence Based Medicine 2011 [25]. We then assessed the risk of bias by the Cochrane risk of bias tool [26] for randomized controlled trials. We evaluated the quality of non-randomized controlled studies by JBI (Joanna Briggs Institute) Critical Appraisal Checklist for Quasi-Experimental Studies [27] Discrepancies between the interpretations was solved by discussion with the senior author (YCL) if consensus was not possible. We summarized the results in a graph of risk for bias and summary table using Reviewer Manager version 5.3.

### 2.5. Statistical Analysis

Only randomized controlled trials were selected for meta-analysis. We chose the primary outcome as the decrease in the IRLS score after injection with BoNT compared with placebo. The results were presented by standardized mean differences (SMD) and 95% confidence intervals. The IRLS score of the intervention group and placebo group 4 weeks after injection was used to analyze the summarized effect size. We set secondary outcome as the adverse event rate in the intervention groups after BoNT administration, which was represented by event rate and 95% confidence intervals. The effect sizes were pooled utilizing the random effect model. We employed the *I^2^* to judge between-study heterogeneity, cutoff values of 50% and 75% were defined as low, moderate, and high [28]. Funnel plots and Egger’s test were adopted to detect the publication bias, and a two tailed *p* < 0.1 indicates statistically significant [29]. Comprehensive Meta-Analysis Software version 3 (Biostat, Englewood, NJ, USA) was used for analysis.

## 3. Results

### 3.1. Study Selection and Description

**Figure 1:**
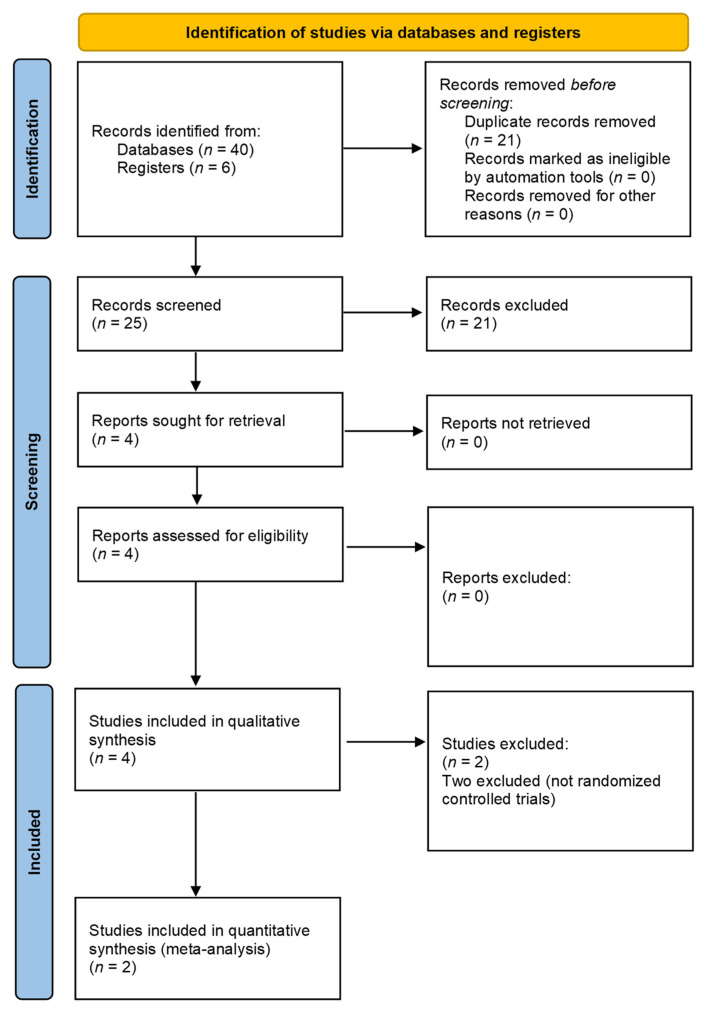
Literature screening process and results.

We identified and removed our last 46 articles from the literature after the first search. Four studies met our criteria of inclusion (Figure 1). We graded the level of evidence of the four articles based on Oxford Centre for Evidence-Based Medicine 2011, two of the articles were level 2 [19,23], and two papers belonged to level 3 [18,22]. We presented the main features of trials included in our systematic review in Table 1.

Among the four included studies, Nahab et al. [23] injected onabotulinumtoxinA to the quadriceps femoris (40 units), tibialis anterior (20 units), gastrocnemius (20 units), and soleus (10 units). Agarwal et al. [18] had 50 units of onabotulinumtoxinA injected into the tibialis anterior in each leg. Mittal et al. [19] injected incobotulinumtoxinA into the tibialis anterior (40 units), gastrocnemius (40 units), and biceps femoris (20 units). Ghorayeb et al. [22] injected intradermally into symptomatic areas (see Table 2 for further details).

After a single treatment session, three studies [18,19,22] showed a short-term improvement in IRLS score, while one trial [23] found no significant improvement. Two articles [18,19,22] identified improvements in the Clinical Global Impression (CGI) rating scales. However, two trials [18,19,22] revealed discrepancies in Visual Analog Scale (VAS) and Epworth Sleepiness Scale (ESS) scores. One study [19] found improvements in quality of life (QoL). Of the four recruited trials, two [18,19] noted no adverse events. Nahab et al. [23] reported that two participants had mild weakness in the injected limb following both saline and BoNT injections. Ghorayeb et al. [22] noticed temporary weakness in the injected limb in seven patients and transient diplopia in one patient.

### 3.2. Risk of Bias Assessment

**Figure 2:**
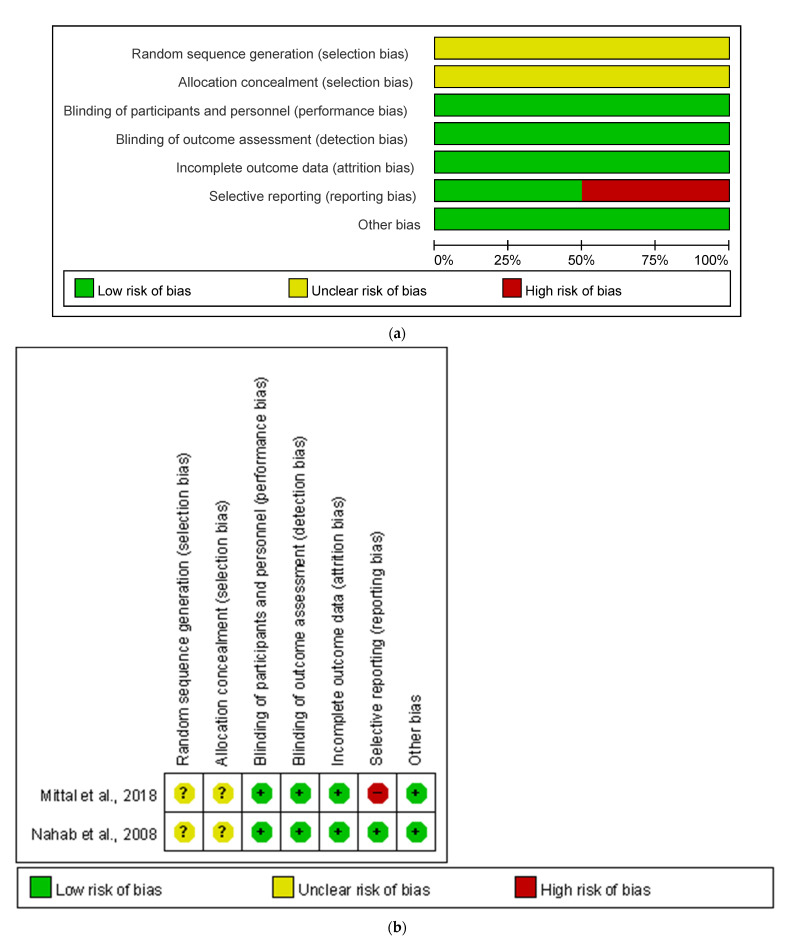

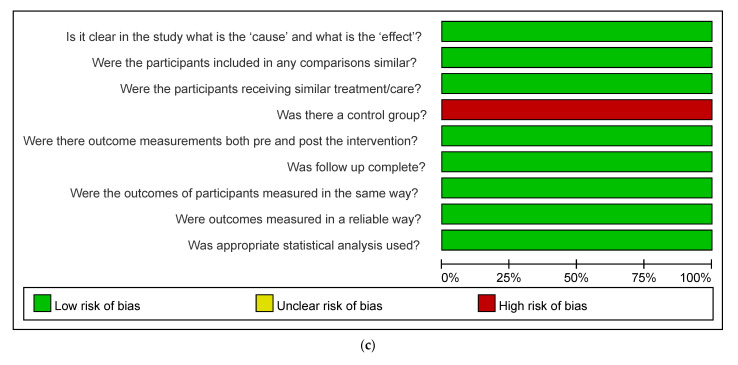

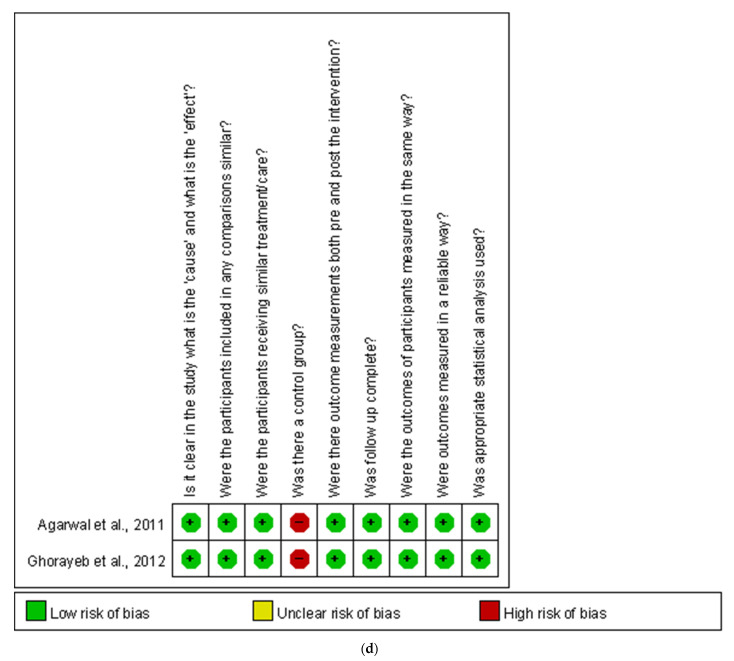
Results of assessments of risk of bias. (**a**) The graph indicates risk of bias of randomized controlled trials, and the questions were from the Cochrane risk of bias tool; (**b**) The table indicates risk of bias of randomized controlled trials, and the questions were from the Cochrane risk of bias tool; (**c**) The graph indicates risk of bias of non-randomized controlled studies, and the questions were from the JBI Critical Appraisal Checklist for Quasi-Experimental Studies; (**d**) The table indicates risk of bias of non-randomized controlled studies, and the questions were from the JBI Critical Appraisal Checklist for Quasi-Experimental Studies.

In the study of Mittal et al. [19], high risk of reporting bias was detected because of selective reporting in patient global impression of change (PGIC) score and Sleep Scale from Medical Outcome Study (MOS), and unclear risk of selection bias was found because the details of sequence generation and allocation were not mentioned in the study. Nahab et al. [23] had an unclear risk of selection bias due to the absence of details of sequence generation and allocation. Both Agarwal et al. and Ghorayeb et al. [18,22] had high risk of bias due to the lack of control groups (Figure 2).

### 3.3. Results of Quantitative Synthesis

**Figure 3:**
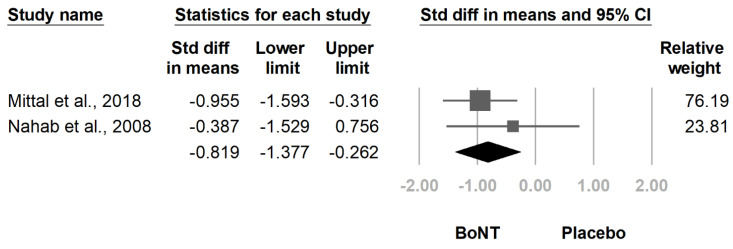
Forest plot of standardized mean differences in International Restless Legs Scale (IRLS) score. Squares indicate effect of individual studies, lines indicate 95% CI, and the diamond indicates the summarized effect size.

**Figure 4:**
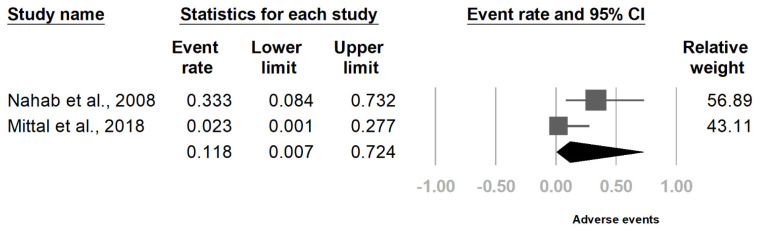
Forrest plot of adverse event rate in the intervention groups after botulinum toxin administration. Squares indicate the rate of individual studies, lines indicate 95% CI, and the diamond indicates the summarized result.

The meta-analysis included two randomized controlled trials [19,23]. Compared with placebo, botulinum toxin injection significantly reduced IRLS score at four weeks after injection (SMD, −0.819, 95% CI, −1.377 to −0.262, *I*^2^ = 0.0%, Figure 3). The rate of adverse events after botulinum toxin injection was 11.8% (95% CI, 0.7–72.4%, *I*^2^ = 70.3%, Figure 4). The primary and secondary outcomes showed low and moderate between-study heterogeneity, respectively. Publication bias was not assessed due to the low number of studies.

## 4. Discussion

Our systemic review and meta-analysis suggested that intramuscular botulinum toxin injection decreased the IRLS score in patients with RLS. Although a few patients complained about adverse events after injection, no serious adverse events were recorded in the articles enrolled in our review.

The results of our meta-analysis corresponded to our initial hypothesis. Our meta-analysis only included randomized controlled trials, which decreased the possible bias caused by placebo effect [30] and ensured the robustness at the evidence level. Furthermore, the low statistical heterogeneity suggested that there might be a correlation between botulinum toxin and an improvement of symptoms in RLS. The fact that no adverse events recorded after BoNT injection may further assure future studies to apply BoNT in patients with RLS. However, these results should be interpreted with caution due to low number of articles included.

According to our review, differences between the results of the included studies may be caused by low statistical power, the dose of BoNT, or the muscles injected. The trial conducted by Nahab et al. [23] had a small sample size of six participants, which might cause a type II error. As for the dose of BoNT and the target muscle of injection, we proposed a hypothesis by comparing the studies included in our review. Although no evidence has revealed a conversion factor between incobotulinumtoxinA and onabotulinumtoxinA in patients with RLS, a 1:1 conversion rate is usually accepted in clinical practice [31]. Under this assumption, Mittal et al. [19] and Agarwal et al. [18] had at least two times the dose of BoNT in the tibialis anterior compared with Nahab et al. [23]. Meanwhile, both Mittal et al. and Agarwal et al. but not Nahab et al. reported a significant decrease in IRLS score after BoNT treatment. This may imply that higher doses of BoNT per muscle especially to the tibialis anterior may be necessary to create positive effects in treatment of patients with RLS, while the total dose may not be the critical factor. This hypothesis may also explain the negative results of Ghorayeb et al. [22], which applied BoNT intradermally. Additionally, a research poster in 2007 concluded that patients with RLS might benefit from 40 to 50 units of onabotulinumtoxinA applied to each tibialis anterior, which corresponded to our hypothesis [20]. However, the pitfall of our hypothesis is that it was drawn by comparing the results of human trials only, and there were insufficient histological nor electrophysiological evidence currently to support the unique roll of tibialis anterior in RLS. Besides, we could not prove this hypothesis quantitatively due to insufficient number of trials included in our review. Further studies are necessary to find the role of tibialis anterior in the pathophysiology of RLS, as well as the optimal dose, site, and commercial form for the administration of BoNT.

We found some pitfalls in the studies included in our review, which may impede the results of our review. First, among the four trials enrolled in this review, three [19,22,23] used the IRLSSG Consensus Criteria for the diagnosis of RLS. However, the fourth article [18] did not mention the criteria that they adopted, which may decrease the generalizability of our conclusion. Second, all of the studies stated that no medication modification aside from the intervention occurred during the trial; however, only one [19] emphasized that non-pharmacological treatments did not change. Non-pharmacological management may affect the symptoms in RLS [6]; therefore, future investigation should exclude such possibility. Third, for investigators using a crossover design, one trial [23] stated that no carryover effect was detected before crossing over, while the other [19] did not mention it. Although Mittal et al. [19] reported that there was no significant difference of all outcomes between botulinum toxin and placebo group at eight weeks after intervention, it would be more adequate to present the outcome measurements before the second phase of trial started. Excluding the possibility of the carryover effect is crucial for crossover studies. Finally, all of the trials administered the botulinum toxin injection once. All of them failed to reveal a treatment effect in IRLS over six weeks. Therefore, consecutive doses may be required for long-term symptom relief. The safety and efficacy of repeat administration of botulinum toxin in patients with RLS requires further investigation.

Several limitations existed in our article of systemic review and meta-analysis. First, there is no standard protocol for botulinum injection in management of patients with RLS. The muscles for injection and dose for individual muscle varied a lot between trials. Hence, although the *I^2^* of our meta-analysis did not recognize a significant heterogeneity, such result should be interpreted with caution. Second, two of the four studies included in this review were single arm trials. Therefore, the absence of a control group made it difficult to exclude any placebo effect. Third, the total number of participants entering the meta-analysis is rather small, which decreases the generalizability of the summarized effect size. Finally, two articles reported adverse events, which were all temporary; however, the low number of total participants may conceal severe adverse events [32].

## 5. Conclusions

The current evidence revealed that botulinum toxin injection may relieve RLS related symptoms. However, decisive conclusions cannot be drawn because of the small number of patients included in our meta-analysis. Many questions remain as to the dose, frequency, and muscle selection for the use of BoNT for the treatment of RLS. No severe adverse events were recognized, but the possibility cannot be excluded due to a low number of participants per study. Randomized controlled trials with more participants are necessary to delineate the potential of botulinum toxin in patients with restless legs syndrome in the future.

## Figures and Tables

**Table 1:** Characteristics of all included studies.

Study	Study Design	Diagnosis Criteria	Age at the Time of BTI (Years)	Mean of Disease Duration (Years)	Enrolled Sample Number (Male/Female)	Post-BTI Follow-Up (Weeks)	Other Treatments Besides BTI
Nahab et al., 2008 [23]	Randomized controlled crossover study	IRLSSG Consensus Criteria	57.7 (8.8)	33.5 (14.4)	6 (3/3)	12	NR
Agarwal et al., 2011 [18]	Non-comparative study	NR	62.75 (9.13)	NR	8 (NR)	12	NR
Ghorayeb et al., 2012 [22]	Non-comparative study	IRLSSG Consensus Criteria	57.6 (14.3)	11 (6–25) ^a^	27 (15/12)	24	NR
Mittal et al., 2018 [19]	Randomized controlled crossover study	IRLSSG Consensus Criteria	BoNT: 64 (13.49) Control: 60.5 (14.75)	NR	BoNT: 14 (8/6) Control: 10 (5/5)	8	NR

Results are given as mean (standard deviation), unless otherwise noted; BTI: botulinum neurotoxin injection; BoNT: botulinum neurotoxin group; Control: control group; IRLSSG: International Restless Legs Syndrome Study Group; NR: not reported. ^a^ median (interquartile range).

**Table 2:** The summarized extracted data from the included studies.

Study	Interval of Continuous Intervention	Commercial Forms	Injection Dose (U)	Dilution Method	Injection Site	Tools for Injection	Outcome Measurement
Nahab et al., 2008 [23]	12 weeks (crossover)	onabotulinumtoxinA	90 U per leg	50 U/mL	Intramuscular	EMG	IRLS score, CGI, AE score ^a^, adverse events
Agarwal et al., 2011 [18]	NR	onabotulinumtoxinA	50 U per leg	100 U/mL	Intramuscular	NR	IRLS score, CGI-S, PGI-S, VAS, ESS; CGI-C, PGI-C; adverse events
Ghorayeb et al., 2012 [22]	NR	abobotulinumtoxinA	500 U–1000 U per patient	250 U/ml	Intradermal	NR	IRLS score, CGI-I; number of responders and duration; adverse events
Mittal et al., 2018 [19]	12 weeks (crossover)	incobotulinumtoxinA	100 U per leg	100 U/ml	Intramuscular	EMG	IRLS score, VAS, QoL, ESS, MOS, PGI-C; adverse events

CGI: Clinical Global Impressions; CGI-I: Clinical Global Impressions- Improvement scale, CGI-S: Clinical Global Impressions-Severity, CGI-C: Clinical Global Impressions- perception of change; EMG: electromyography; ESS: Epworth Sleepiness Scale; IRLS: International Restless Legs Syndrome Study Group rating scale; MOS: Sleep Scale from Medical Outcome Study; NR: not reported; PGI-C: Patient Global Impression–perception of change; PGI-S: Patient Global Impression-Severity; QoL: quality of life; U: unit; VAS: Visual Analog Scale. ^a^ AE scores: adverse effects, rating from 0 (no symptoms) to 10 (severe symptoms) of the presence of weakness, pain, swelling, and redness based on the preceding two weeks after injection.

## Data Availability

No new data were created in this study.

## Supplementary Materials

The following are available online at https://www.mdpi.com/article/10.3390/healthcare9111538/s1, File S1: Search strategies for systematic review and meta-analysis.
