# Supplementary material for: Effectiveness and Safety of Botulinum Toxin Type A in Treatment of Restless Legs Syndrome: A Systematic Review and Meta-Analysis
Source: Healthcare (Basel). 2021 Nov 11;9(11):1538. doi: 10.3390/healthcare9111538 (PMC8623507; doi:10.3390/healthcare9111538)
Supplement: Supplementary file 1 [file healthcare-09-01538-s001.zip › healthcare-1392366-supplementary.pdf]

File S1. Search strategies for systematic review and meta-analysis.

The search was performed on June 12, 2021.

PUBMED Results: 13

#8 Search: #3 AND #6 Filters: English 13
#7 Search: #3 AND #6 13
#6 Search: #4 OR #5 22541
#5 Search: "botulinum toxins"[mesh terms] 16830
#4 Search: "botulinum toxin" 22541
#3 Search: #1 OR #2 5482
#2 Search: "restless legs syndrome "[mesh terms] 3858
#1 Search: restless leg syndrome 5482

Cochrane Central Register of Controlled Trials Results: 6

#8 #3 AND #6 in Trials 6
#7 #3 AND #6 6
#6 #4 OR #5 4260
#5 botulinum toxin* 4260
#4 MeSH descriptor: [Botulinum Toxins] explode all trees 1940
#3 #1 OR #2 552
#2 restless leg syndrome 301
#1 MeSH descriptor: [Restless Legs Syndrome] explode all trees 348

Web of Science Results: 27

#4 (#1 AND #2) AND LANGUAGE: (English) 27
#3 #1 AND #2 27
#2 TS=(botulinum toxin*) 24235
#1 TS=(restless leg syndrome) 7592
